# Liquid Chromatography/Mass Spectrometry based serum metabolomics study on recurrent abortion women with antiphospholipid syndrome

**DOI:** 10.1371/journal.pone.0225463

**Published:** 2019-11-21

**Authors:** Lili Zhang, Ying Li, Xiuying Lin, Chunshu Jia, Xiaowei Yu

**Affiliations:** 1 Department of Ultrasonography, First Hospital, Jilin University, Changchun, China; 2 Department of Neonatology, First Hospital, Jilin University, Changchun, China; 3 Center for Reproductive Medicine, Jilin Province People's Hospital, Changchun, China; 4 Centre for Reproductive Medicine, Centre for Prenatal Diagnosis, First Hospital, Jilin University, Changchun, China; International University of Health and Welfare, School of Medicine, JAPAN

## Abstract

**Objective:**

The antiphospholipid syndrome (APS) is an important cause of acquired thromboembolic complications and pregnancy morbidity. The pathogenic mechanisms that damage the fetal–maternal unit and cause abnormal placental development are incompletely understood in APS patients. Liquid Chromatography/Mass Spectrometry (LC/MS) based metabolomics are applied for the mechanism of disease and further supporting the research of diagnosis and management in recent years. The aim of this research was to investigate the difference of serum metabolic profiles in recurrent abortion women with APS and healthy women to explore the mechanism of this disease.

**Methods:**

Serum samples of 25 recurrent abortion women with APS and 25 healthy women were collected and analyzed by LC/MS in this study. Potential biomarkers were discovered by multivariate statistical analysis and then identified based on analysis results.

**Results:**

Totally, we identified five biomarkers that involved in different metabolic pathway such as purine metabolism, amino acid metabolism and tyrosine metabolism. These biomarkers showed different roles in disease development.

**Conclusion:**

Metabolomics was proved as a powerful tool in understanding the mechanism of recurrent abortion caused by APS.

## Introduction

The antiphospholipid syndrome (APS) is a chronic autoimmune disease with rheumatic inflammatory, which can induce state of hypercoagulation. APS includes two clinical manifestations: vascular thrombosis and pregnancy complications [1]. APS is marked by the presence of antibodies in blood. The phospholipid-binding proteins, not the phospholipid, were recognized and attacked by antibodies [2]. APS was diagnosed according to the clinical symptoms of pregnancy morbidity, arterial thrombosis or venous and on laboratory tests consisting in the detection of antibodies such as lupus anticoagulant (LA) or which directed against β2-glycoprotein I (aβ2GPI) and cardiolipin (aCL) [3]. Pregnancy complications are challenging problems in APS patients. For pregnant women with APS, the primary treatment is focused to prevent thrombosis; however, it is partially successful only [4]. It is necessary to understand more about the mechanism of APS and its effect on pregnant women.

Multiple studies have shown that the relationship between immune response and metabolism. Metabolomic analysis aims at a comprehensive characterization of biological samples [5]. The information of altered metabolic profile between APS patients and healthy control can be used for diagnosis and therapy of disease. It is required that the analytical methods could generate comprehensive metabolic profiles from complicated sample matrix so that the comprehensive metabolome coverage can be achieved. Nuclear magnetic resonance (NMR) and mass spectrometry are two analytical tools that have been well applied in metabolomics-based studies [6, 7]. Wide dynamic range and chemical diversity coverage are the main advantages of LC/MS. It is very suitable for untargeted metabolomics study.

In this study, LC/MS based metabolomics method was applied to investigate the metabolic profile change between recurrent abortion women with APS and healthy women. This study is aim to identify the potential biomarkers for APS and explore the biological pathways change. Furthermore, the study results could provide a new insight on the diagnosis and management of pregnant women with APS.

## Material and methods

### Study subjects

25 recurrent abortion women with APS and 25 healthy women as healthy control were recruited from Center for Reproductive Medicine, Jilin Province People's Hospital, from Jan 2017 to Aug 2018. All the tests were performed in Sep 2018. The study had been approved by the Ethics Committee of Jilin Province People's Hospital. Prior to inclusion in this study, informed consent was got from all participants. All the authors had no access to information that could identify individual participants during or after data collection.

APS patients had been previously diagnosed based on the updated Sapporo classification criteria [8], whereas the control group comprised healthy people without any major illnesses or abortion history. People who already had or recently had any medical conditions, and who had the history of drug addiction, or who had history of alcohol abuse should be excluded from this study.

In 1999, the proposed Sapporo classification criteria for APS were first published [9], and then, it was updated in the 11th International Congress on Antiphospholipid Antibodies, 2006 in Sydney [8]. To meet a diagnosis of APS, the patient need to have both the clinical criteria and laboratory criteria. To investigate and understand the complicated mechanism of APS, we chose the women who meet both criteria below for the disease group: (a) ultrasound or direct examination of the fetus reported normal fetal morphology, and then one or more unexplained deaths of normal fetus happened at or over the 10th week of gestation; (b) Serum anticardiolipin (aCL) antibody of IgG presented in medium or high titer based on ELISA results measured in two or more occasions, over 12 weeks interval. Meanwhile, the control group are the women who had normal history of pregnancy and negative results of antiphospholipid-antibody. All the samples are collected 6 months after abortion or childbirth to prevent any potential effect on metabolic profile during pregnancy.

Serum samples were collected and stored at −80°C until analysis.

### Serum anticardiolipin (aCL) antibody of IgG testing

Using Bio-Rad Anti-Cardiolipin IgG EIA Kit #425–2000 to test the Serum anticardiolipin (aCL) antibody of IgG of clinical samples. Testing procedure followed the instruction of the Kit.

### Sample preparation

Serum samples were melted at room temperature. 100μL serum were added 400μL acetonitrile and vertex 30 seconds, then put it at room temperature for 10 minutes, centrifuge for 10 minutes in 12000rpm, using 0.22μm filter to filter the supernatant. The filtered samples were finally analyzed by LC/MS.

### Metabolomics analysis

Agilent 1200 rapid resolution liquid chromatography (RRLC) coupled with Agilent 6520 Q-TOF mass spectrometer (Agilent Technologies, USA) was used for the LC/MS analysis. Sample volume was 10μL. Column: Agilent Eclipse Plus C18, 3.5μm, 2.1mm×150mm. Column temperature: 30°C.

The LC condition was as below, Solvent A: water with 0.1% formic acid, v/v; Solvent B: acetonitrile with 0.1% formic acid, v/v. Flow rate: 0.3 mL/min. Solvent gradient: 0–5 min, 95%-80% solvent A; 5–10 min, 80%-60% solvent A; 10–15 min, 60%-2% solvent A; 15–16 min 2%-0% solvent A; and 16–17 min, 0% solvent A. 30 min for post run.

Mass spectrometry condition: Electrospray ionization (ESI) source; Positive mode: gas temperature, 350°C; drying gas, 9L/min; nebulizer, 40psig; fragmentor, 150V; skimmer, 60V; capillary, 3500V. Negative mode: gas temperature, 350°C; drying gas, 9L/min; nebulizer, 40psig; fragmentor, 150V; skimmer, 60V; capillary, 3500V. Centroid mode for data collection, from *m/z* 50 to 1000.

### Pattern recognition and pathway analysis

Data normalization was performed and then imported the data files into the statistic software (Mass Profiler Professional, Agilent Technologies in USA). After further processing, using T-test and principal component analysis (PCA) for the comparison of the metabolites profiles between APS group and healthy group. Biomarker identification used public databases in internet such as HMDB (http://www.hmdb.ca/) and KEGG (http://www.kegg.com/).

### Thyroid function testing

Serum thyroid function test was performed to evaluate the thyroid function of the APS and healthy women. Total T3 (TT3), Total T4 (TT4), Free T3 (FT3), Free T4 (FT4), reverse T3 (rT3) and thyroid stimulating hormone (TSH) were tested using ThyroChek thyroid testing kit.

## Results

### Antiphospholipid antibodies (aPL) profile of 25 APS patients

The Serum aCL, Serum anti-b2GPI, IgG aCL and IgG anti-b2GPI profile of the 25 APS patients are shown in Table 1.

**Table 1:** aPLs profile of 25 patients.

Patient No.	Serum aCL (GPLU) 112.8–146.2	Serum anti-b2GPI (GBU)68.3–30.1	IgG aCL (GPLU) 46.3–86.5	IgG anti-b2GPI (GBU)50.8–26.2
1	114.5	62.1	48.3	30.1
2	132.3	54.3	81.2	49.8
3	144.5	60.2	56.4	28.8
4	130.8	30.1	76.2	35.9
5	142.3	68.3	66.1	26.2
6	146.2	45.3	48.2	48.5
7	112.8	52.4	46.3	50.8
8	118.9	39.1	82.1	33.1
9	136.4	47.8	86.5	29.7
10	140.7	55.3	58.3	50.2
11	119.4	49.8	69.2	28.9
12	128.3	43.2	77.3	44.3
13	125.6	50.7	72.5	39.4
14	131.9	33.8	82.3	36.5
15	121.4	44.5	55.4	42.1
16	113.2	50.2	60.1	37.3
17	142.1	54.5	73.5	44.4
18	133.4	47.9	64.7	35.7
19	127.3	50.2	71.2	38.9
20	134.3	39.1	78.2	40.4
21	129.3	57.1	49.8	43.3
22	122.7	48.5	53.2	48.6
23	127.3	51.1	58.7	31.3
24	128.6	44.4	66.9	34.6
25	133.3	50.1	77.4	33.7

### Metabolic profiling analysis

**Fig 1:**
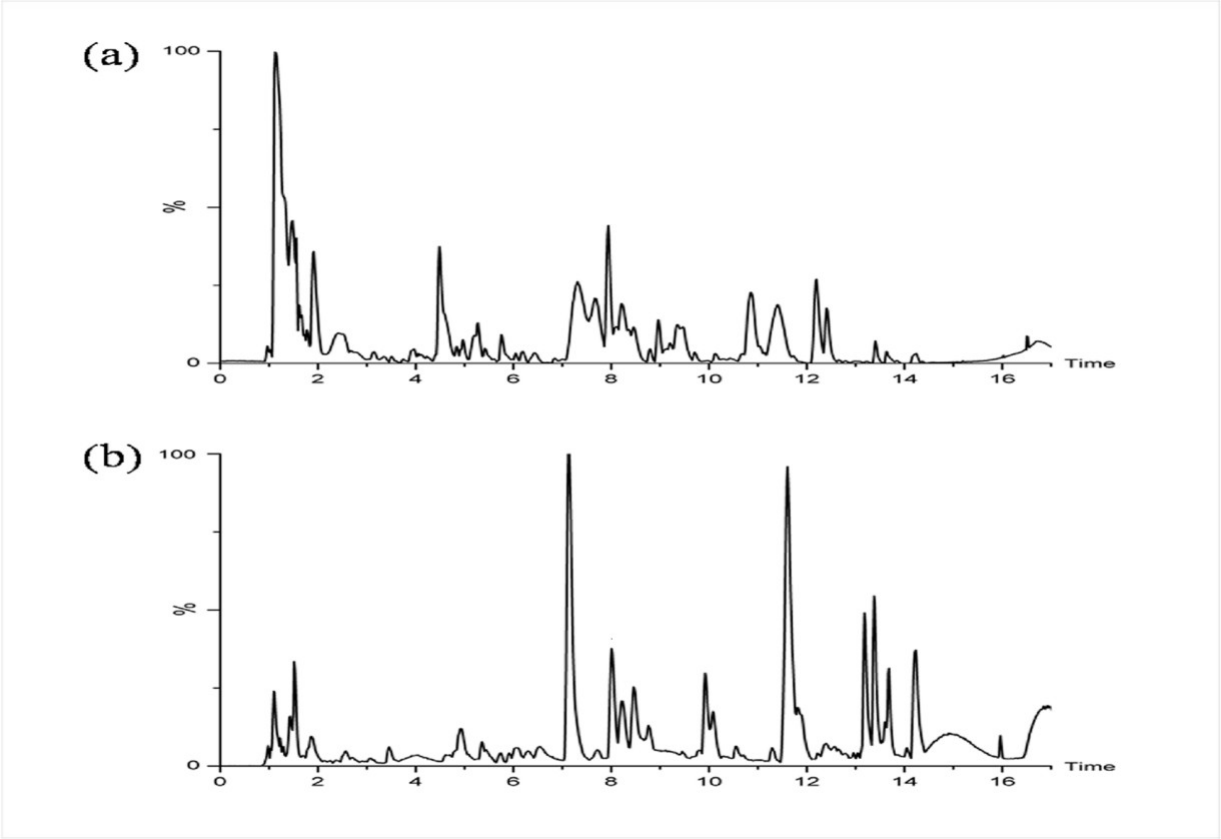
(a) The positive and (b) negative ion base peak intensity (BPI) chromatograms of the serum sample from an APS patient.

Using Agilent RRLC/Q-TOF/MS to analyze the serum metabolites profiles of the samples collected from APS and control groups. The representative base peak intensity (BPI) chromatograms of the serum sample of an APS patient in positive and negative mode were shown in Fig 1, respectively.

Method validation was performed using quality control (QC) sample, which was prepared by mixing 10μL for each serum from all the collected samples in this study. QC sample was analyzed to evaluate the system stability following every five injection. Five ions in positive mode (*m/z* 315.2368, 259.1455, 169.5847, 469.3568 and 366.5248) and five ions in negative mode (*m/z* 176.3257, 138.2659, 256.3257, 359.8746 and 459.3681) were selected for the extraction of the ion chromatographic peaks. The system stability (RSDs %) of *m/z*, peak areas and retention times of the ten selected ions were 0.02–0.14%, 0.0003–0.0007% and 5.9–8.5%, respectively. During the analysis, the QC results achieve perfect system stability, reproducibility of chromatographic separation and mass measurement that demonstrated the analysis results are reliable.

**Fig 2:**
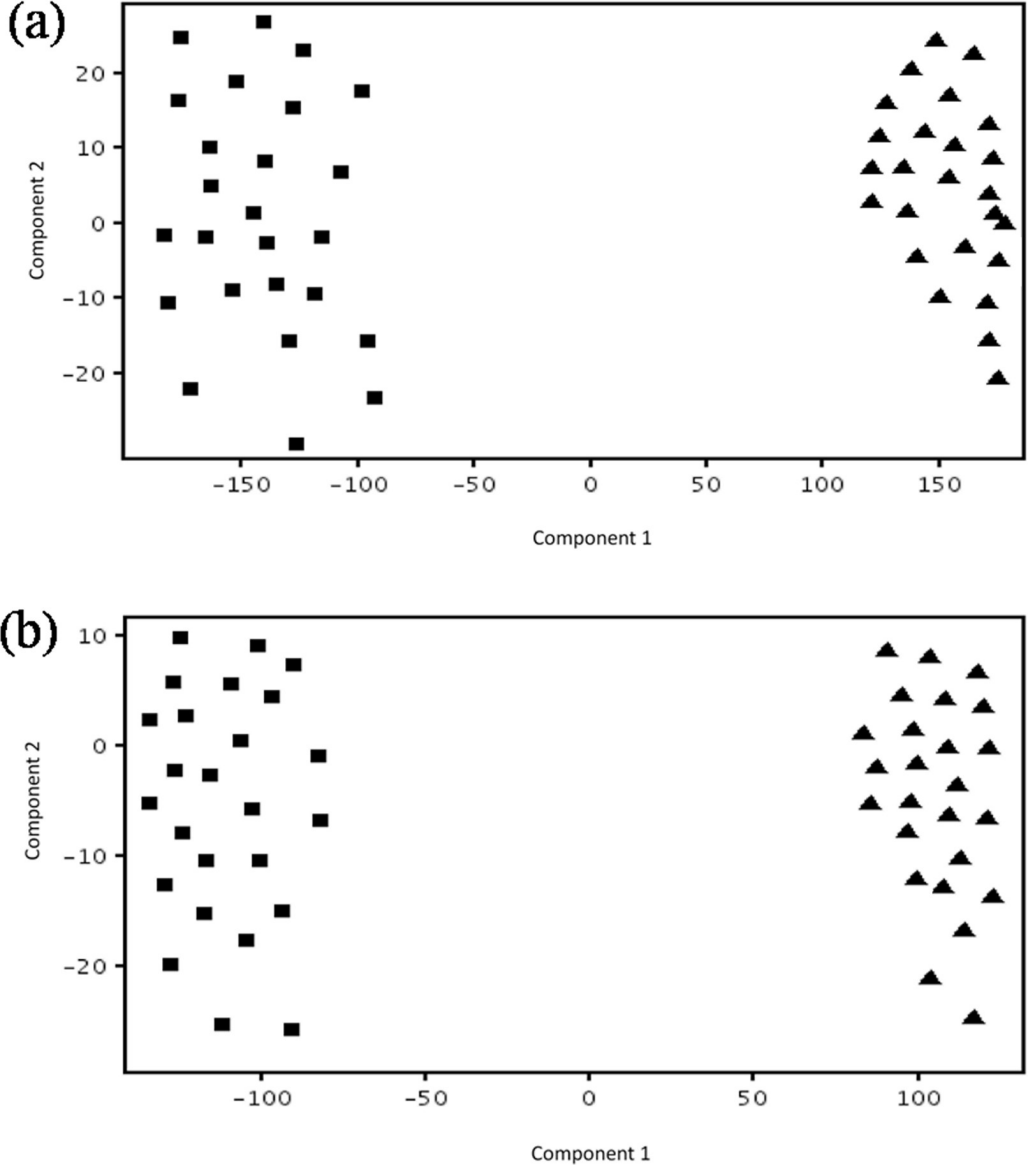
Score plots (a) in positive ion mode and (b) negative ion mode from PCA model classifying APS (■) and control group (▲).

PCA was performed for the evaluation of the changed metabolic profile between recurrent abortion women with APS and healthy control. PCA score plots classifying the recurrent abortion women with APS and control group was shown in Fig 2(A) and 2(B). It can be seen that the two groups are clearly separated no matter it is positive or negative modes. The clear separation showed the significantly different metabolic profile between recurrent abortion women with APS and control group.

**Fig 3:**
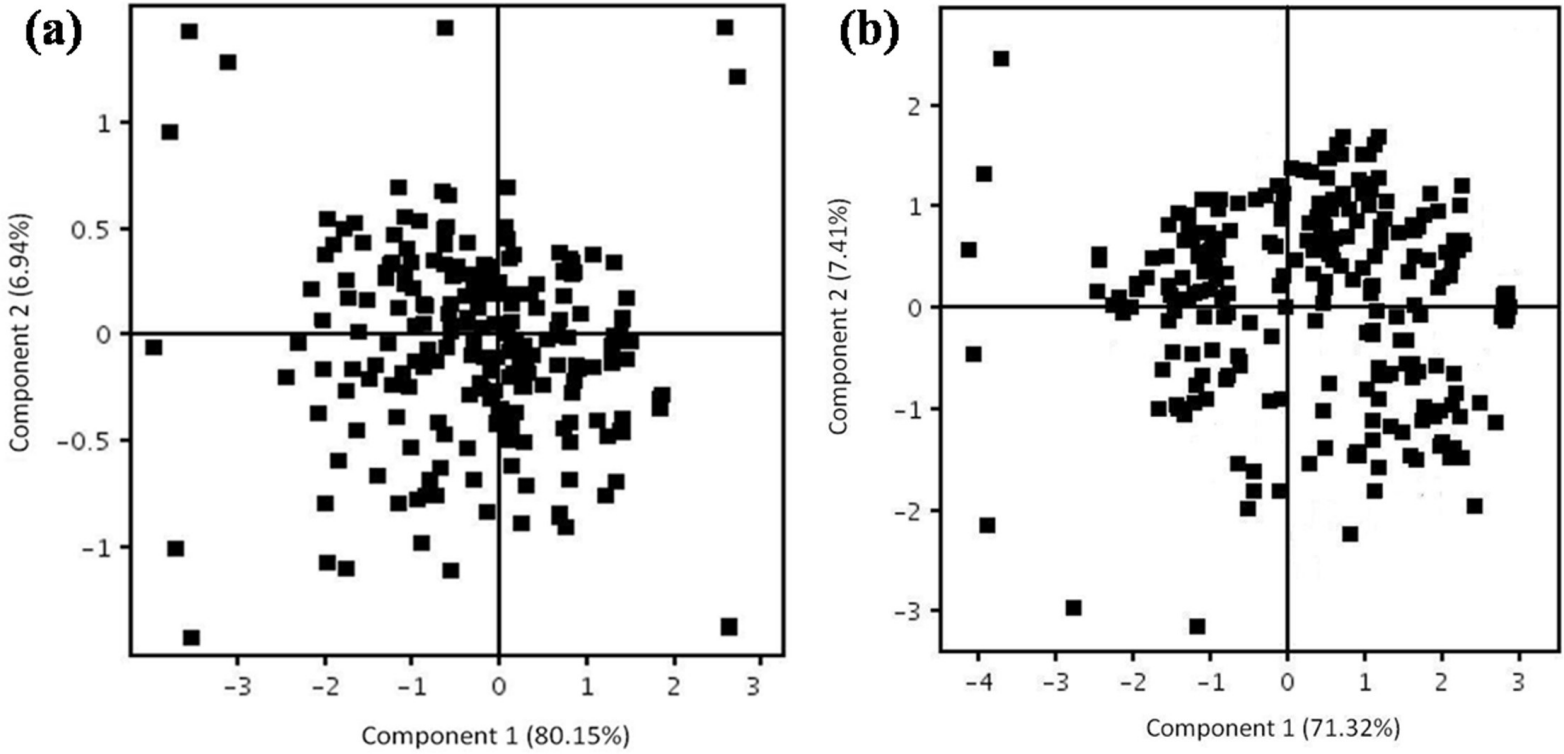
Loading plots (a) in positive ion mode and (b) negative ion mode from PCA model classifying APS and control group.

Fig 3(A) and 3(B), the loading plots for biomarker discovery, showed the metabolites ions in positive and negative ion modes. The ions that are furthest from the origin of the plots were identified as the potential biomarkers. These biomarkers make most contribution to the separation of recurrent abortion women with APS and healthy control.

### Biomarker identification

Independent test provide the *p* values of different ions. There are totally twelve ions showed significant differences in the two groups. However, due to the limitation of the database, we can identify five potential biomarkers. The accurate molecular ion masses were used for the potential composition calculation and the MS/MS data were collected for structure deduction. The database search were used to further confirm the identification results. HMDB and KEGG were used for identification. At last, the validation of the identification results were performed by comparing the MS and MS/MS data of the available standards. The identification results and the change trends of the metabolites are listed in Table 2.

**Table 2:** Identification results and the change trends of the biomarkers.

Metabolites	Theory (*m/z*)	Observed (*m/z*)	P-values	Value of APS (mg/dL)	CV(%) of APS	Value of control (mg/dL)	CV(%) of control	APS vs control	pathway
Positive ion mode									
Uric Acid	169.0361	169.0355	0.0044	6.23	20	4.81	22	Increase	Purine metabolism
Arginine	175.1195	175.1185	0.0021	56.43	18	40.43	17	Increase	Amino acid metabolism
Creatine	132.0773	132.0776	0.0042	15.11	15	23.12	19	Decrease	Amino acid metabolism
Creatinine	114.0667	114.0672	0.0028	1.54	22	2.84	20	Decrease	Amino acid metabolism
Negative ion mode									
Thyroxine	775.6789	775.6798	0.0014	28.34	18	39.31	21	Decrease	Tyrosine metabolism

### Thyroid function testing

Table 3 showed the results of thyroid function testing and it indicates TT3, TT4, FT4 and TSH were significantly decreased in APS patients.

**Table 3:** Thyroid function test results of APS and control.

	APS	Control
TT3	2.2± 0.5nmol/L*	2.4± 0.4nmol/L
TT4	34.5± 16.8nmol/L*	88.5± 18.9nmol/L
FT4	8.7± 2.4pmol/L*	18.6± 5.4pmol/L
FT3	7.8± 1.9pmol/L	8.3± 2.4pmol/L
rT3	0.4±0.2nmol/L	0.5± 0.2nmol/L
TSH	0.5± 0.3mIU/L*	2.1± 0.9mIU/L

*P<0.05

## Discussion

The metabolite profile changes were studied between APS patients and healthy controls. RRLC-QTOF-MS were used for comprehensive metabolomics analysis and provide the overall metabolites change. With multivariate statistical analysis, the recurrent abortion women with APS group and control group were clearly separated. From the loading plots of the PCA, the potential biomarkers were showed in both positive and negative ion modes and contributed to the separation of recurrent abortion women with APS and control group. The *p* values of independent test showed that twelve compounds are significantly different between the two groups.

These biomarkers indicated the involvement of the metabolic pathways of purine metabolism, amino acid metabolism and tyrosine metabolism in APS development. The metabolic pathway analysis confirmed the fact that the APS is related to multiple pathogenesis mechanism.

Uric acid is generated by purine nucleotides breakdown in metabolic process. Increased uric acid level in serum will cause arthrolithiasis and be associated with many medical conditions including diabetes and the kidney stones formatted by ammonium acid urate. The elevated uric acid in blood of patients who have pregnancy complications was considered to be renal dysfunction. However, there is new evidence indicated that elevation of uric acid is caused by the placenta. Previous research found that antiphospholipid antibodies (aPL) can increase the production of trophoblast uric acid. Meanwhile, the uric acid play a role as endogenous secondary signals to activate the trophoblast inflammation by stimulating the inflammasome in trophoblasts [10]. Another study showed that in patients with mild-to-moderate SLE, uric acid elevated at midgestation that correlated with preterm birth [11]. Given that uric acid and related biologic pathway play an important role in activating inflammasome in placenta in response to aPL, it may be beneficial to inhibit the uric acid pathway in APS patients.

Arginine is a semiessential amino acid that can be used for protein biosynthesis. The synthesis mainly occurs from citrulline both in the liver and in kidney. Nitric oxide (NO)’s immediate precursor is arginine. NO act as the second messenger that was considered as an important signaling molecule, and NO is an intercellular messenger regulating vasodilation. At the same time, in the immune system, NO has the functions of response reaction to infection. Immune cells show the ability of NO synthesis. Considering that NO is potent immunomodulators, arginine metabolism play significant roles in physiological and pathological conditions [12]. L-arginine significantly delayed the thrombus formation in a coronary occlusion canine model [13]. Administration of L-arginine stimulated the thrombus lysis and inhibited platelet aggregations in the coronary arteries. These studies indicated that arginine might have positive effect on APS patients. The increased arginine in APS patients might be induced by the physiological condition of APS and be positive on the body.

Thyroid function testing results shows TT3, TT4, FT4 and TSH were significantly decreased in APS patients. Triiodothyronine (T3) and thyroxine (T4) are two tyrosine-based thyroid hormones, which are produced by the thyroid gland and then released in the body.

The two hormones are mainly responsible for metabolism regulation. Thyroid hormones are critical in many target tissues such as the brain and skeleton for their growth and maturation. Maternal thyroxine is crucial in fetal development due to it supplies thyroid hormone-dependent tissues in critical periods during the first trimester of pregnancy [14]. Transthyretin are well known that it plays an important role in the transport of thyroxine and retinol from maternal to fetal circulation. In addition, transthyretin was identified as the protein biomarker in women with pregnancy morbidity associated with APS [15]. These discoveries indicated that thyroid dysfunction might be associated with APS in pregnant women and contribute to pregnancy loss.

Creatine’s function is adenosine triphosphate (ATP) recycling. ATP is the cell energy currency, which is primarily existed in brain and muscle tissue in vertebrates. Serum creatinine testing is a common used test for the indication of renal function. In addition, the low levels of creatinine in the body could be a sign that the liver or muscles are not working well. Creatine and creatinine metabolism is associated with a variety of diseases [16]. Some animal experiments demonstrated that when creatine was given as a supplement in the diet of pregnant women, it provided protection on the diaphragm, fetal brain and kidney against hypoxic insult at term [17–19]. These results suggested us to consider the possibility of giving creatine as supplement in the management of APS women.

In present study, the LC-MS based metabolomics method was applied to analyze the metabolic profile changes between recurrent abortion women with APS and control subjects. The results of identification of five potential biomarkers have shown that the APS is closely related to purine metabolism, amino acid metabolism and tyrosine metabolism. Furthermore, the potential biomarkers showed the metabolism disorder of APS and may be further investigated for diagnosis and therapy. It is proved that the LC/MS metabolomics method is useful for APS mechanism study. The application of metabolomics can provide more metabolic information to support the research on diagnosis and therapy of recurrent abortion caused by APS.

## Supporting information

S1 File Compounds detected in negative ion model. (XLSX)

S2 File Compounds detected in positive ion model. (XLSX)
